# Human papillomavirus E1 proteins inhibit RIG-I/MDA5-MAVS, TLR3-TRIF, cGAS-STING, and JAK-STAT signaling pathways to evade innate antiviral immunity

**DOI:** 10.3389/fimmu.2025.1549766

**Published:** 2025-04-22

**Authors:** Jin-Xin Li, Jing Zhang, Cheng-Hao Li, Yun-Fang Li, Hui-Min Chen, Tao Li, Qing Zhang, Bei-Hua Kong, Pei-Hui Wang

**Affiliations:** ^1^ Department of Infectious Disease and Hepatology, The Second Hospital of Shandong University, Cheeloo College of Medicine, Shandong University, Jinan, Shandong, China; ^2^ Key Laboratory for Experimental Teratology of Ministry of Education and Advanced Medical Research Institute, Cheeloo College of Medicine, Shandong University, Jinan, Shandong, China; ^3^ Department of Obstetrics and Gynecology, Qilu Hospital, Shandong University, Jinan, China; ^4^ Gynecologic Oncology Key Laboratory of Shandong Province, Qilu Hospital, Shandong University, Jinan, China

**Keywords:** HPV E1, innate immunity, RIG-I/MDA5-MAVS, cGAS-STING, JAK-STAT, immune evasion, interferon

## Abstract

Human papillomavirus (HPV) is a major etiological agent of both malignant and benign lesions, with high-risk types, such as HPV16 and HPV18, being strongly linked to cervical cancer, while low-risk types like HPV11 are associated with benign conditions. While viral proteins such as E6 and E7 are well-established regulators of immune evasion, the role of E1 in modulating the host antiviral responses remains insufficiently characterized. This study investigates the immunomodulatory functions of HPV16 and HPV11 E1 in suppressing innate antiviral immune signaling pathways. Through a combination of RT-qPCR and luciferase reporter assays, we demonstrate that E1 suppresses the production of interferons and interferon-stimulated genes triggered by viral infections and the activation of RIG-I/MDA5-MAVS, TLR3-TRIF, cGAS-STING, and JAK-STAT pathways. Co-immunoprecipitation assays reveal that E1 interacts directly with key signaling molecules within these pathways. E1 also impairs TBK1 and IRF3 phosphorylation and obstructs the nuclear translocation of IRF3, thereby broadly suppressing IFN responses. Additionally, E1 disrupts the JAK-STAT pathway by binding STAT1, which prevents the assembly and nuclear localization of the ISGF3 complex containing STAT1, STAT2, and IRF9, thereby further diminishing antiviral response. These findings establish E1 as a pivotal regulator of immune evasion and suggest its potential as a novel therapeutic target to enhance antiviral immunity in HPV-associated diseases.

## 1 Introduction

Human papillomaviruses (HPVs) represent a highly diverse group of double-stranded DNA viruses that primarily infect epithelial cells. Over 200 HPV types have been identified, broadly categorized into high-risk (HR) and low-risk (LR) types according to their oncogenic potential (1, 2). HR HPVs, particularly HPV16 and HPV18, are predominantly associated with malignancies such as cervical, anogenital, and oropharyngeal cancers (3, 4). HPV16 is the most prevalent type, accounting for nearly 50% of cervical cancers and a considerable proportion of HPV-related head and neck squamous cell carcinomas (HNSCCs) (5, 6). Although HPV18 is less frequent, it remains a significant contributor to oncogenesis (4). Conversely, LR HPVs, such as HPV11, are mainly associated with benign lesions, notably genital warts and respiratory papillomatosis (7). Despite their lower oncogenic potential, LR HPVs still pose a considerable health burden due to persistent infections, which result in chronic inflammation and epithelial alterations (8). HPV’s ability to evade host immune responses is pivotal for establishing persistent infections, enabling long-term viral survival and immune evasion. Key viral proteins, particularly E6 and E7 in HR HPVs, have evolved diverse strategies to inhibit host immune pathways, with a focus on innate immunity—the first line of defense against viral infections (9, 10). This immune evasion underpins the virus’s capacity to persist asymptomatically for years, complicating disease management and prevention.

Upon infection, the host’s innate immune system detects viruses through pattern recognition receptors (PRRs), which identify viral components and trigger antiviral responses (11). Several innate immune pathways mediate the recognition of virus infections, including Toll-like receptors (TLRs) (12), RIG-I-like receptors (RLRs) (13), and the cyclic GMP-AMP synthase (cGAS)-stimulator of interferon genes (STING) pathway (14, 15). TLR3 recognizes viral double-stranded RNA (dsRNA) in endosomes and activates TRIF, resulting in the production of type I interferons (IFN-α/β) (16, 17). Similarly, RIG-I and MDA5 recognize viral RNA in the cytoplasm and trigger downstream immune responses via the adaptor protein mitochondrial antiviral signaling protein (MAVS) (18, 19). Upon detecting cytosolic DNA, cGAS generates cyclic GMP-AMP (cGAMP), which binds to and activates STING (20). These pathways converge on TANK-binding kinase 1 (TBK1), which phosphorylates interferon regulatory factor 3 (IRF3). Once phosphorylated, IRF3 translocates to the nucleus, initiating the transcription of type I IFNs (21). These type I IFNs bind to IFNα receptor (IFNAR) on neighboring cells, triggering the JAK-STAT pathway (22), which involves the phosphorylation of Janus kinases (JAK1 and JAK2) and the transcription factors STAT1 and STAT2 (23). Once phosphorylated, STAT1 and STAT2 form a trimeric complex with IRF9, known as the interferon-stimulated gene factor 3 (ISGF3) complex. The ISGF3 complex translocates to the nucleus, where it binds to interferon-stimulated gene (ISG) promoter regions, triggering their transcription and amplifying the antiviral response (24, 25).

The HPV genome encodes early viral proteins (E1, E2, E4, E5, E6, and E7) and late structural proteins (L1 and L2) (26, 27). Among these, E6 and E7 are well-established oncogenic drivers, facilitating malignant transformation by disrupting critical regulatory pathways in host cells (28). HPV also employs other early proteins, such as E5, to disrupt immune signaling (29). For instance, E5 impairs TLR3-TRIF signaling, while HPV16 E6 downregulates TLR3 expression (30, 31). Additionally, E6 and E7 interfere with the RIG-I/MDA5-MAVS and cGAS-STING pathways, with E6 inhibiting RIG-I ubiquitination and E7 impairing STING function (32, 33). Together, these viral proteins disrupt innate immune signaling, enabling HPVs to evade host immune detection and establish persistent infections that drive tumorigenesis.

HPV E1 primarily facilitates viral replication, acting as a helicase that unwinds viral DNA at the replication origin (34, 35). E1 comprises multiple functional domains, including an N-terminal domain for nucleocytoplasmic transport regulation, a DNA binding domain, an oligomerization domain for hexamer formation, and a C-terminal helicase domain (34). E1 is recruited to the viral origin by E2, forming the E1-E2-ori complex that is crucial for viral replication (36, 37). Additionally, E1 has been associated with processes such as DNA damage induction and cell cycle regulation (38, 39). While emerging evidence indicates that E1 may modulate innate immune responses, research on its immune regulatory roles remains limited (40, 41). Unraveling E1’s contribution to viral immune evasion could uncover novel mechanisms facilitating viral persistence and pathogenesis.

In this study, we examined the role of HPV11 and HPV16 E1 proteins in modulating host innate immunity. Our findings reveal that E1 proteins from both HPV11 and HPV16 suppress multiple antiviral signaling pathways, including RIG-I/MDA5-MAVS, TLR3-TRIF, cGAS-STING, and JAK-STAT. This inhibition targets specific signaling molecules, including MAVS, TRIF, TBK1, and STAT1, leading to diminished IFN production and ISG expression. These findings offer new insights into HPV immune evasion mechanisms, revealing a broader role for E1 in promoting viral persistence than previously recognized.

## 2 Materials and methods

### 2.1 Cell culture

HEK293T, HEK293TT, HeLa, Vero, and L929 cells (American Type Culture Collection, ATCC) were cultured at 37°C in a 5% CO_2_ incubator using Dulbecco’s Modified Eagle’s Medium (DMEM; Gibco, USA), supplemented with 10% fetal bovine serum (FBS; Gibco) and 1% penicillin-streptomycin (Gibco). The culture medium was replaced every 2-3 days, and cells were passaged when they reached 70-80% confluence.

### 2.2 Transfection

HEK293T and HEK293TT cells were transiently transfected with plasmids or poly(I:C) (1 μg/mL) using polyethylenimine (PEI) Max (Polysciences, USA) following the manufacturer’s protocol, while HeLa cells were transfected with Lipofectamine 2000 (Invitrogen, USA). Both transfection reagents were applied at optimized ratios according to the manufacturer’s guidelines.

### 2.3 Plasmids

Plasmids encoding RIG-I, RIG-IN (active RIG-I), MDA5, MAVS, STING, TBK1, IKKϵ, IRF3-5D (active IRF3 mutant), and TRIF were prepared as described in prior studies (42–44). Briefly, RIG-I, MDA5, and TBK1 were cloned into the expression vector pXJ2-Flag; IKKϵ and TRIF were cloned into pXJ2-Myc; RIG-IN (active RIG-I) was cloned into pcDNA6B-Myc; IRF3-5D (an active IRF3 mutant) was cloned into pXJ2-HA; and MAVS was cloned into pCMV-HA. For the expression of STAT1, STAT2, and IRF9, the corresponding genes were cloned into the pXJ2-Flag, pXJ2-HA, or pXJ2-V5 vectors. Luciferase reporter plasmid pGL3-IFN-β-Luc was constructed by cloning the IFN-β promoter regions into the pGL3 empty vector measuring IFN-β promoter activities. Luciferase reporter plasmid pGL4.20-ISRE-Luc used for measuring ISG promoter activity was constructed by insert the interferon-stimulated response element (5´-GAAACTGAAACTGAAACTGAAACTGAAACTGAAACTGAAACTGAAACTGAAACTGAAACT-3´) into the multiple cloning region of pGL4.20 vector (Promega, USA). Organelle marker plasmids pDsRed2-Mito (mitochondria), pDsRed2-ER (endoplasmic reticulum), and pEYFP-Golgi (Golgi apparatus) were acquired from Clontech (USA) for co-localization studies. Plasmids containing the complete HPV16 or HPV11 genomes were obtained from ATCC. Codon optimized E1 DNA fragments (**Supplementary Materials**) of HPV11 and HPV16 were synthesized (General Biol, China) and cloned into pXJ2-Flag and pXJ2-Myc vectors. All constructs were sequence-verified prior to use.

### 2.4 Antibodies and reagents

The antibodies, including mouse anti-GAPDH (3B3), mouse anti-Myc (19C2), goat anti-mouse IgG-FITC, goat anti-rabbit IgG-FITC, goat anti-mouse IgG-Cy3, and goat anti-rabbit IgG-Cy3, were obtained from Abmart (China). Mouse anti-IRF3 (CY5779), mouse anti-TBK1 (CY5145), and mouse anti-Lamin B1 (AB0054) antibodies were purchased from Abways (USA). Rabbit anti-pIRF3 (4D46), rabbit anti-pTBK1 (D52C2), and rabbit anti-Myc (71D10) antibodies were purchased from Cell signaling Technology (USA). Mouse anti-Flag M2 antibody was obtained from Sigma-Aldrich (USA). Rabbit anti-Flag antibody was acquired from Immunoway (USA). Peroxidase-conjugated secondary antibody, anti-mouse (AB0102) and anti-rabbit (AB1010), were obtained from Abways. Fluorescence secondary antibodies, including Alexa Fluor 488 goat anti-rabbit IgG, Alexa Fluor 594 goat anti-rabbit IgG, Alexa Fluor 488 goat anti-mouse IgG, and Alexa Fluor 594 goat anti-mouse IgG were acquired from Beyotime (China). Anti-Flag magnetic beads were obtained from Abmart.

### 2.5 Quantitative real-time

Total RNA was extracted using TRIzol reagent (Invitrogen) and treated with gRNA wiper (Vazyme, China) to eliminate genomic DNA. Reverse transcription was performed using the HiScript II 1st Strand cDNA Synthesis Kit (Vazyme). SYBR Green-based RT-qPCR was carried out using a Roche LightCycler 96 system. GAPDH was used as the reference gene, and relative expression levels were calculated using the 2^-ΔΔCt^ method. Each reaction was performed in triplicate.

### 2.6 Luciferase reporter assays

HEK293T cells (~4×10^4^ per well) were seeded into 96-well plates and co-transfected with firefly luciferase reporter plasmids, pathway activator plasmids, and an internal control Renilla luciferase plasmid. A total of 120 ng plasmid DNA was transfected per well, with pcDNA6B serving as an empty vector to equalize the total DNA amount. After 30 hours, cells were lysed, and relative luciferase activity was determined by normalizing firefly luciferase activity to Renilla luciferase activity.

### 2.7 Co-immunoprecipitation and immunoblotting

HEK293T cells (3×10^6^ per flask) were seeded into T25 flasks and transfected with the appropriate plasmids. After 36-48 hours, cells were washed with PBS and lysed in ice-cold lysis buffer (150 mM NaCl, 1% NP-40, 50 mM EDTA, and 50 mM Tris-HCl, pH7.4) supplemented with protease and phosphatase inhibitors (Sigma-Aldrich, USA) for co-immunoprecipitation assays. Cell lysates were collected by centrifugation at 13,000 g for 15 minutes at 4°C, and protein concentrations were measured using a BCA Protein Assay Kit (Beyotime). For input analysis, one-tenth of each lysate was mixed with 5 × SDS loading buffer and heated at 100°C for 15 minutes. The remaining supernatant was incubated with anti-Flag magnetic beads (Sigma-Aldrich) overnight at 4°C. Immunoprecipitates were washed four times with lysis buffer, resuspended in 2× SDS loading buffer, and heated at 100°C for 10 minutes. For immunoblotting, cell lysates were mixed with 5 × SDS loading buffer and heated at 100°C for 10 minutes. Proteins were resolved by SDS-PAGE and transferred onto PVDF membranes (Millipore, USA). Membranes were blocked in 5% (wt/vol) nonfat milk in TBST and then incubated with the specified primary antibodies followed by the secondary antibodies. Detection was carried out using the SuperSignal Chemiluminescent ECL Reagent Kit (Beyotime).

### 2.8 Immunofluorescence

HEK293T cells (1×10^5^ per well) and HeLa cells (3×10^4^ per well) were seeded onto coverslips for culturing. Cells were transfected with plasmids for 20 hours and then stimulated with specified virus. Six hours post-stimulation, cells were fixed with 4% paraformaldehyde, permeabilized with 0.2% Triton X-100, blocked, and incubated overnight at 4°C with primary and secondary antibodies. Slides were mounted using DAPI-containing medium (Beyotime), and images were acquired using a Zeiss LSM900 confocal microscope. The immunofluorescence images were quantified using ImageJ.

### 2.9 Nuclear and cytoplasmic protein extraction

Nuclear and cytoplasmic protein fractions were isolated using the Nuclear and Cytoplasmic Protein Extraction Kit (Beyotime, P0027) following the manufacturer’s instructions. In brief, cells were harvested, washed with ice-cold PBS, and then incubated on ice for 20 minutes in cytoplasmic extraction buffer supplemented with protease and phosphatase inhibitors, with intermittent mixing. NP-40 was added to the cytoplasmic extraction buffer, and the lysates were centrifuged at 6,000 g for 10 minutes at room temperature to isolate the cytoplasmic fraction. The pellet was resuspended in nuclear extraction buffer (P0027-3) and incubated on ice for 30 minutes with occasional vortexing. The nuclear fraction was collected by centrifugation at 13,000 g for 15 minutes. Purity of the nuclear and cytoplasmic fractions was verified by immunoblotting, using Lamin B1 as a nuclear marker and GAPDH as a cytoplasmic marker.

### 2.10 Viral infections

Murine coronavirus mouse hepatitis virus-A59 (MHV-A59), herpes simplex virus 1 (HSV1), vesicular stomatitis virus (VSV) expressing enhanced green fluorescent protein (eGFP), Sendai virus (SeV), and HPV16 virions were used to infect target cells, following established protocols (45, 46). Cells were washed with prewarmed serum-free DMEM and incubated with virus diluted in DMEM at the specified multiplicity of infection (MOI) for 1–2 hours. After infection, the virus-containing supernatant was removed, and cells were replenished with fresh complete DMEM. HPV16 virions were generated using a transient transfection protocol as described previously (46). HEK 293TT cells were seeded in 10-cm dishes one day prior to transfection and transfected with plasmids encoding HPV16 L1 and L2 capsid proteins, along with the HPV16 genome. After 48 hours of incubation at 37°C, cells were harvested, lysed using a custom lysis buffer, and concentrated via PEG8000 precipitation. The packaged virions were used to infect HeLa cells in subsequent experiments.

### 2.11 Viral plaque assays

To quantify viral titers, Vero cells (1.2 × 10^5^ per well) were infected with serially diluted VSV-eGFP or HSV1 for 1 hour. After infection, cells were washed with PBS and cultured in DMEM supplemented with 0.5% agar and 2% FBS for 24 hours. Cells were fixed with a methanol: ethanol mixture (1:1) for 30 minutes, and the agarose-medium overlay was carefully removed. Fixed cells were stained with 0.05% crystal violet, and plaques were counted to determine viral titers.

### 2.12 Statistics

Statistical analyses were performed using a two-tailed unpaired Student’s *t*-test in GraphPad Prism 9.0. The results are presented as mean ± standard deviation (SD) from three independent experiments. Statistical significance was defined as *p < 0.05, ** *p* < 0.01, *** *p* < 0.001, ****p < 0.0001.

## 3 Results

### 3.1 HPV E1 inhibits IFN responses induced by virus infections

**Figure 1:**
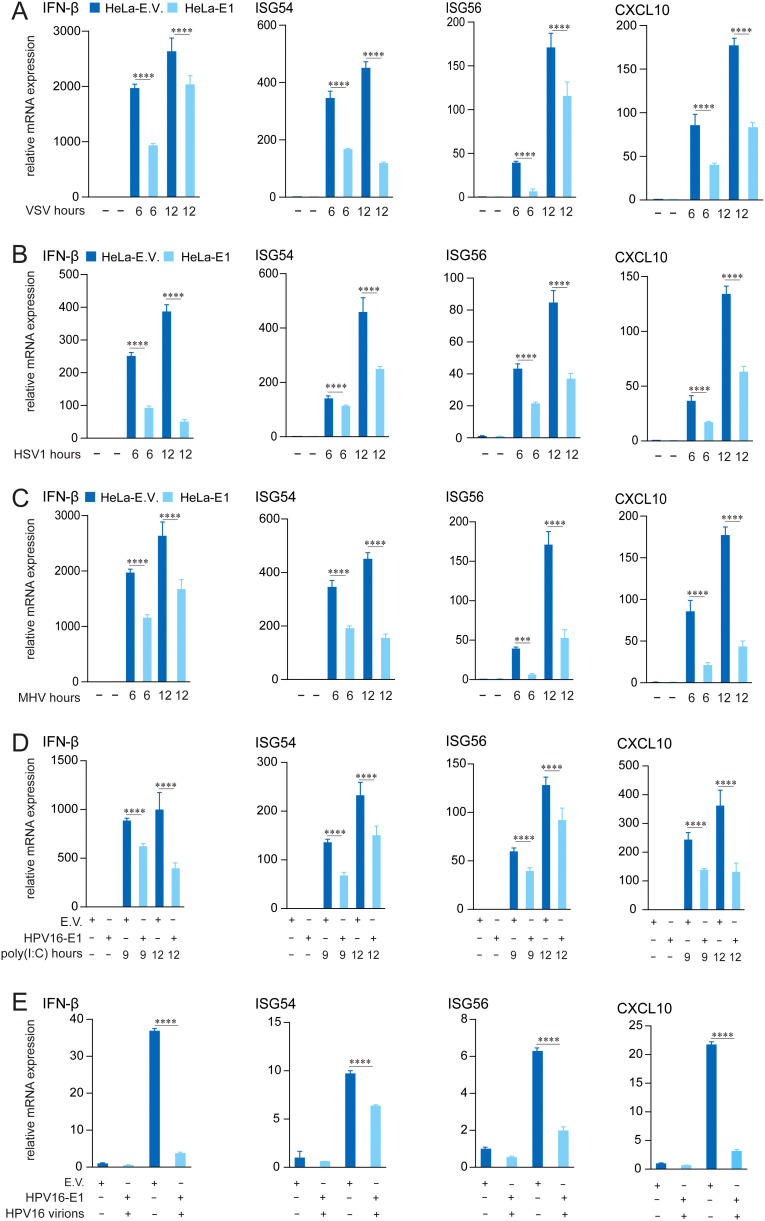
E1 Suppresses IFN and ISG activation induced by multiple stimuli. **(A-C)** HeLa cells stably expressing HPV16 E1 or an empty vector were infected with VSV (MOI=0.1) **(A)**, HSV1 (MOI=1) **(B)**, and MHV-A59 (MOI=0.2) **(C)** for 6 and 12 hours, respectively. **(D)** HEK293T cells transfected with HPV16 E1 or an empty vector were stimulated with poly(I:C) for 9 and 12 hours. **(E)** HeLa-E1 and HeLa-E.V. cells were infected with HPV16 virions for 24 hours. mRNA levels of IFN-β, CXCL10, ISG54, and ISG56 were quantified by RT-qPCR. Statistical significance was analyzed using Student’s t-test (*** p < 0.001, **** p < 0.0001). E.V, empty vector.

Persistent infection with HR HPV16 is a well-established risk factor for cervical cancer and other conditions, largely attributed to the virus’s capacity to evade host immune responses. Previous studies have demonstrated that HPV non-structural proteins, such as E5, E6, and E7, suppress host immune response (32, 41, 47–49). To evaluate whether HPV16 E1 inhibits virus-induced immune responses, we generated HeLa cells stably expressing HPV16 E1 or an empty vector control. These cells were infected with the RNA viruses (VSV and MHV) and the DNA virus HSV1. RT-qPCR analysis revealed significant reductions in mRNA levels of IFN-β, ISG54, ISG56, and CXCL10 in HPV16 E1-expressing cells compared to controls across multiple post-infection time points (**Figures 1A–C**). These results were further validated in HEK293T cells transiently transfected with HPV16 E1 or an empty vector. Following poly(I:C) stimulation, HPV16 E1-expressing cells exhibited marked reductions in IFN-β, ISG54, ISG56, and CXCL10 mRNA levels (**Figure 1D**). Additionally, HeLa cells expressing HPV16 E1 and infected with HPV16 virions showed significant reductions in IFN-β and ISG expression compared to control cells (**Figure 1E**). These findings indicate that HPV16 E1 broadly suppresses type I IFN and ISG responses induced by RNA viruses, DNA viruses, and synthetic viral RNA analogs.

### 3.2 HPV E1 facilitates virus replication

**Figure 2:**
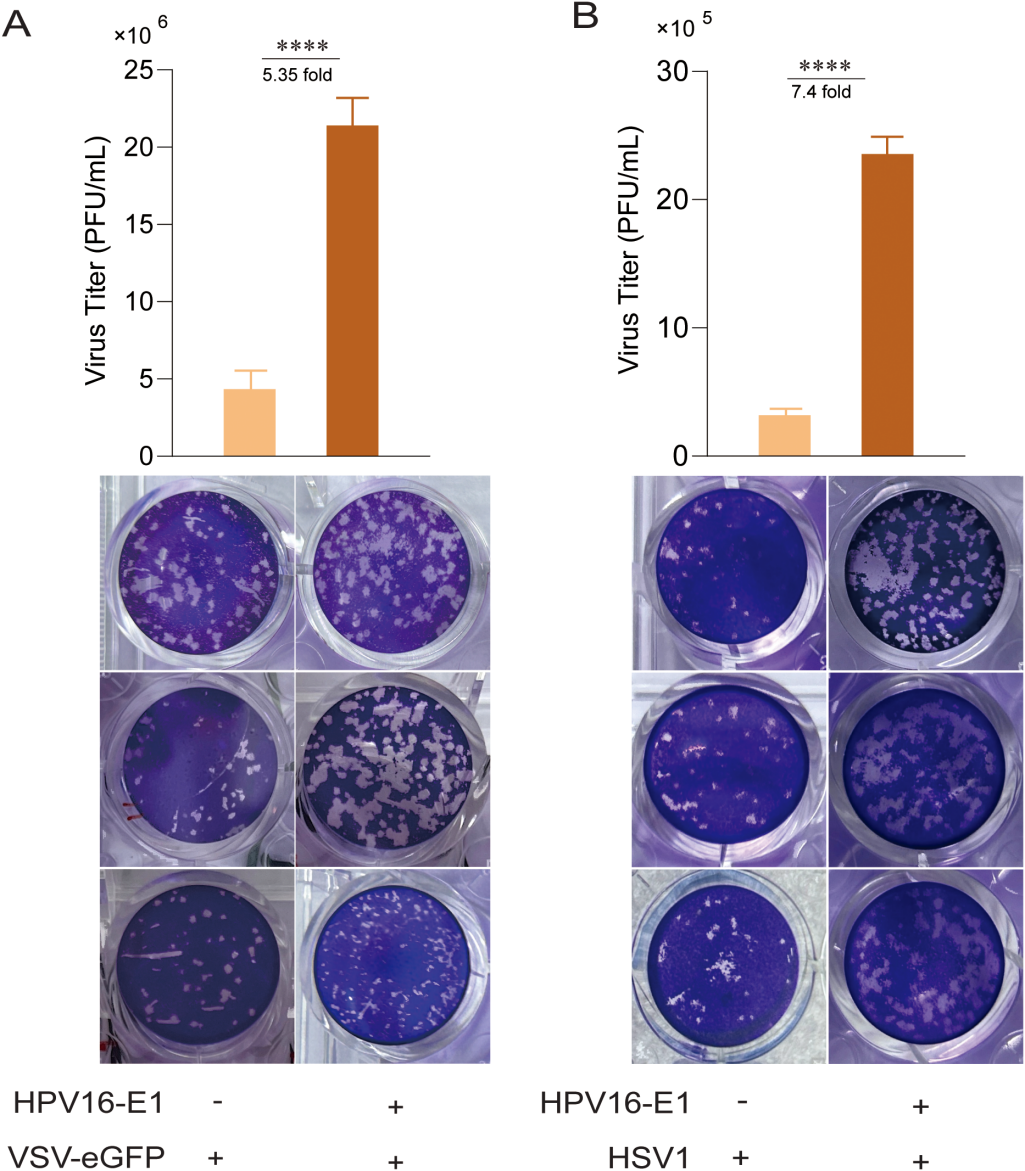
E1 enhances viral replication. HEK293T cells transfected with HPV16 E1 or an empty vector were infected with VSV-eGFP (MOI = 0.1) **(A)** and HSV1 (MOI = 1) **(B)** for 24-36 hours. Viral titers in the culture supernatants were measured using plaque assays. Statistical significance was analyzed using Student’s t-test (**** p < 0.0001).

Since HPV16 E1 suppresses IFN response, we investigated its impact on viral replication. HEK293T cells expressing HPV16 E1 were infected with VSV-eGFP and HSV1, and viral titers in culture supernatants were quantified using plaque assays. Results showed that HPV16 E1 expression significantly enhanced the viral titers of both VSV-eGFP and HSV1 compared to control cells (**Figures 2A, B**). These findings suggest that HPV16 E1 promotes viral replication, likely through IFN response suppression.

### 3.3 HPV E1 inhibits RIG-I/MDA5-MAVS, TLR3-TRIF, and cGAS-STING pathways

**Figure 3:**
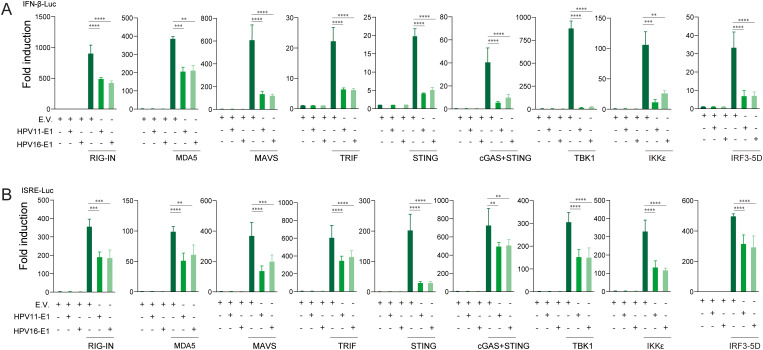
E1 inhibits IFN and ISRE luciferase reporters activated by RIG-I/MDA5-MAVS, TLR3-TRIF, and cGAS-STING pathways. HEK293T cells were co-transfected with HPV11 or HPV16 E1 plasmids and pathway activators, along with the IFNβ-Luc **(A)** or the ISRE-Luc reporters **(B)**. Luciferase activity, measured 30 hours post-transfection, reflects the productions of type ι IFNs and ISGs. pRL-TK was used as an internal control, and each experiment was conducted in three biological replicates. Statistical significance is indicated in the figure (** p < 0.01, *** p < 0.001, **** p < 0.0001).

**Figure 4:**
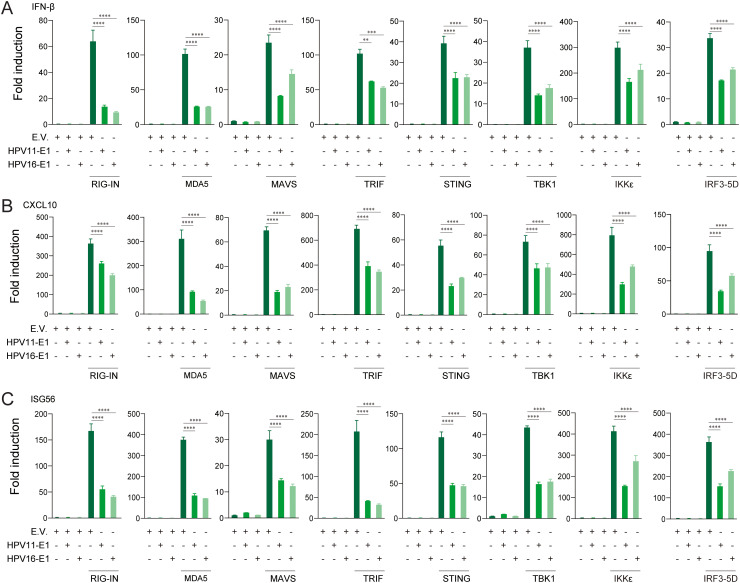
E1 impairs IFN and ISG production induced by RIG-I/MDA5-MAVS, TLR3-TRIF, and cGAS-STING pathways. HEK293T cells transfected with HPV11 E1, HPV16 E1, or an empty vector were co-transfected with pathway activators for 24 hours. mRNA levels of IFN-β **(A)**, CXCL10 **(B)**, and ISG56 **(C)** were quantified by RT-qPCR. The data represent one representative experiment (n = 3 biological replicates) and are presented as mean ± SD. Statistical significance is indicated in the figure (** p < 0.01, *** p < 0.001, **** p < 0.0001).

To identify the innate immune pathways suppressed by HPV E1, HEK293T cells were co-transfected with HPV16 or HPV11 E1 and plasmids encoding key activators of the RIG-I/MDA5-MAVS, TLR3-TRIF, and cGAS-STING pathways. Luciferase reporter assays revealed that E1 expression significantly suppressed IFN-β and ISRE promoter activation induced by each pathway activator, including RIG-IN, RIG-I, MDA5, MAVS, TBK1, IKKϵ, STING, TRIF, and IRF3-5D (**Figures 3A, B**). RT-qPCR analysis further confirmed that HPV E1 significantly reduced mRNA levels of IFN-β, CXCL10, and ISG56 in cells co-transfected with pathway activators (**Figures 4A–C**). These findings suggest that HPV E1 broadly inhibits antiviral signaling across multiple PRR pathways.

### 3.4 HPV E1 interacts with key proteins of innate immune signaling pathways

**Figure 5:**
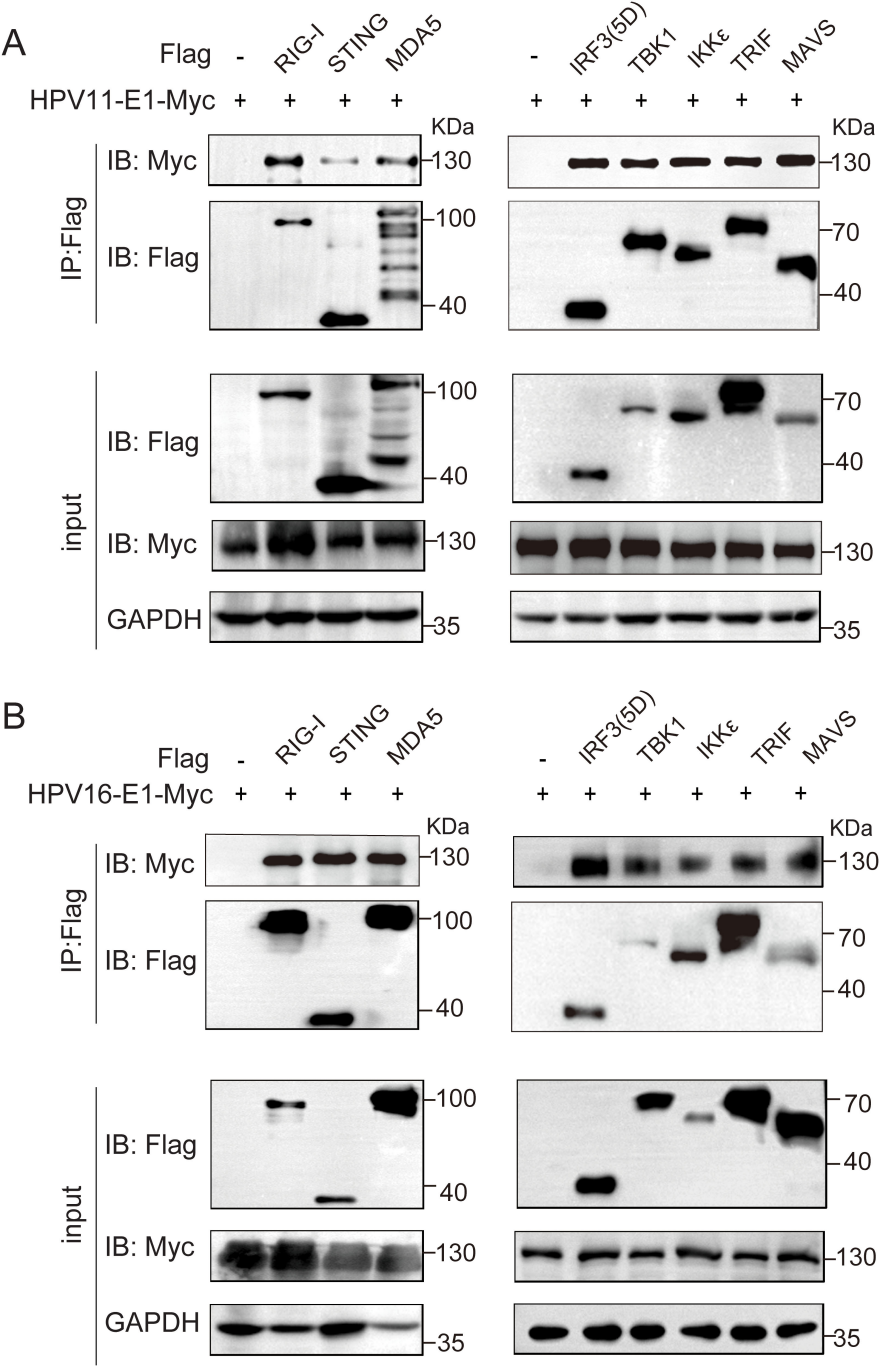
E1 Interacts with multiple immune signaling molecules. HEK293T cells co-transfected with HPV11 E1 **(A)** or HPV16 E1 **(B)** and signaling molecule plasmids were lysed 36-48 hours post-transfection. Protein interactions were assessed using co-immunoprecipitation and Western blot analyses.

**Figure 6:**
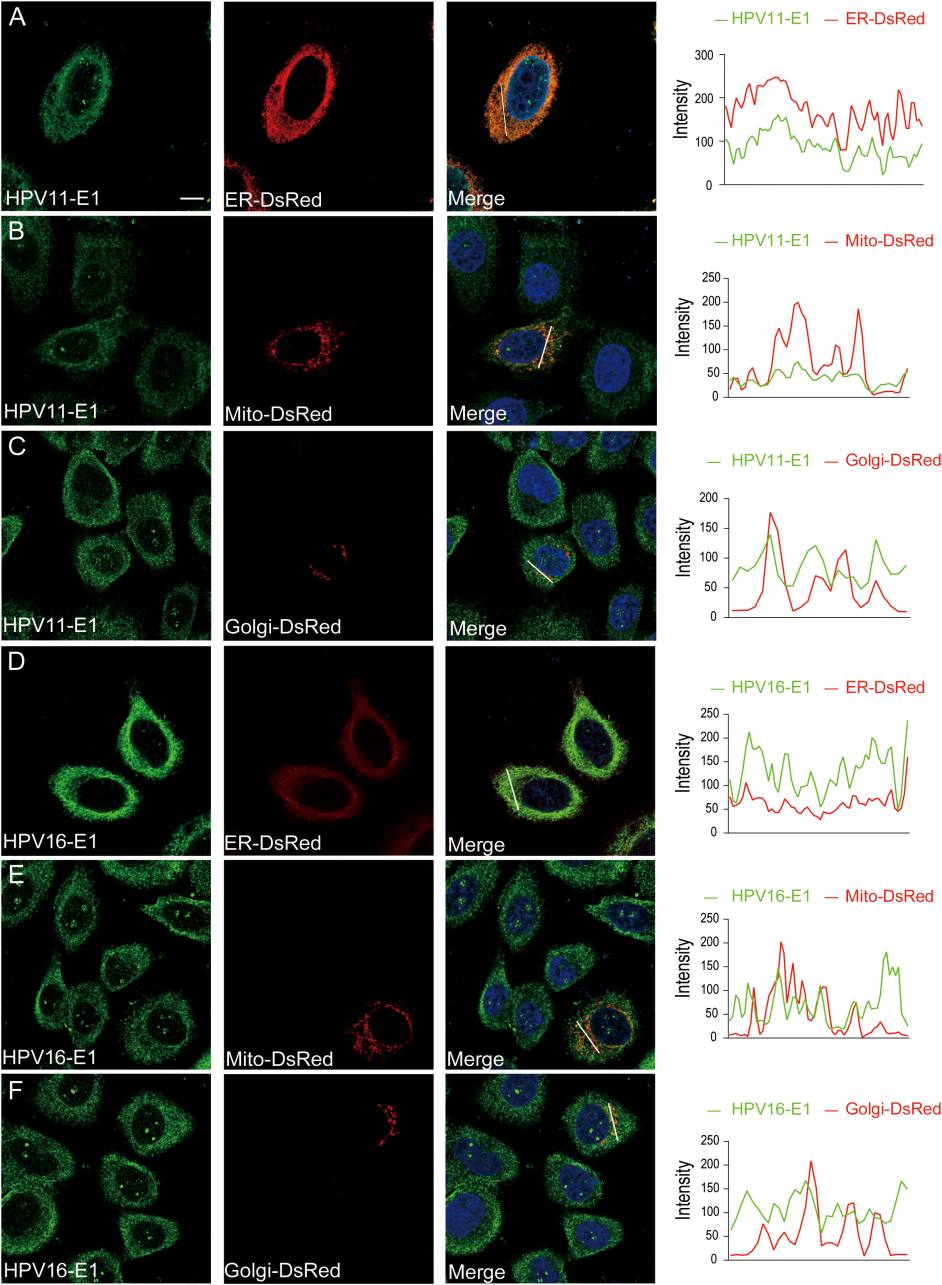
Subcellular localization of E1. HeLa cells were transfected with HPV11 E1 **(A-C)** or HPV16 E1 **(D-F)** and organelle markers for ER (pDsRed2-ER), mitochondria (pDsRed2-Mito), or Golgi (pEYFP-Golgi). After 20 hours, cells were fixed, blocked, incubated with primary and fluorescence-labeled secondary antibodies, and visualized using confocal microscopy. Nuclei were stained with DAPI (blue). Scale bar, 10 μm.

**Figure 7:**
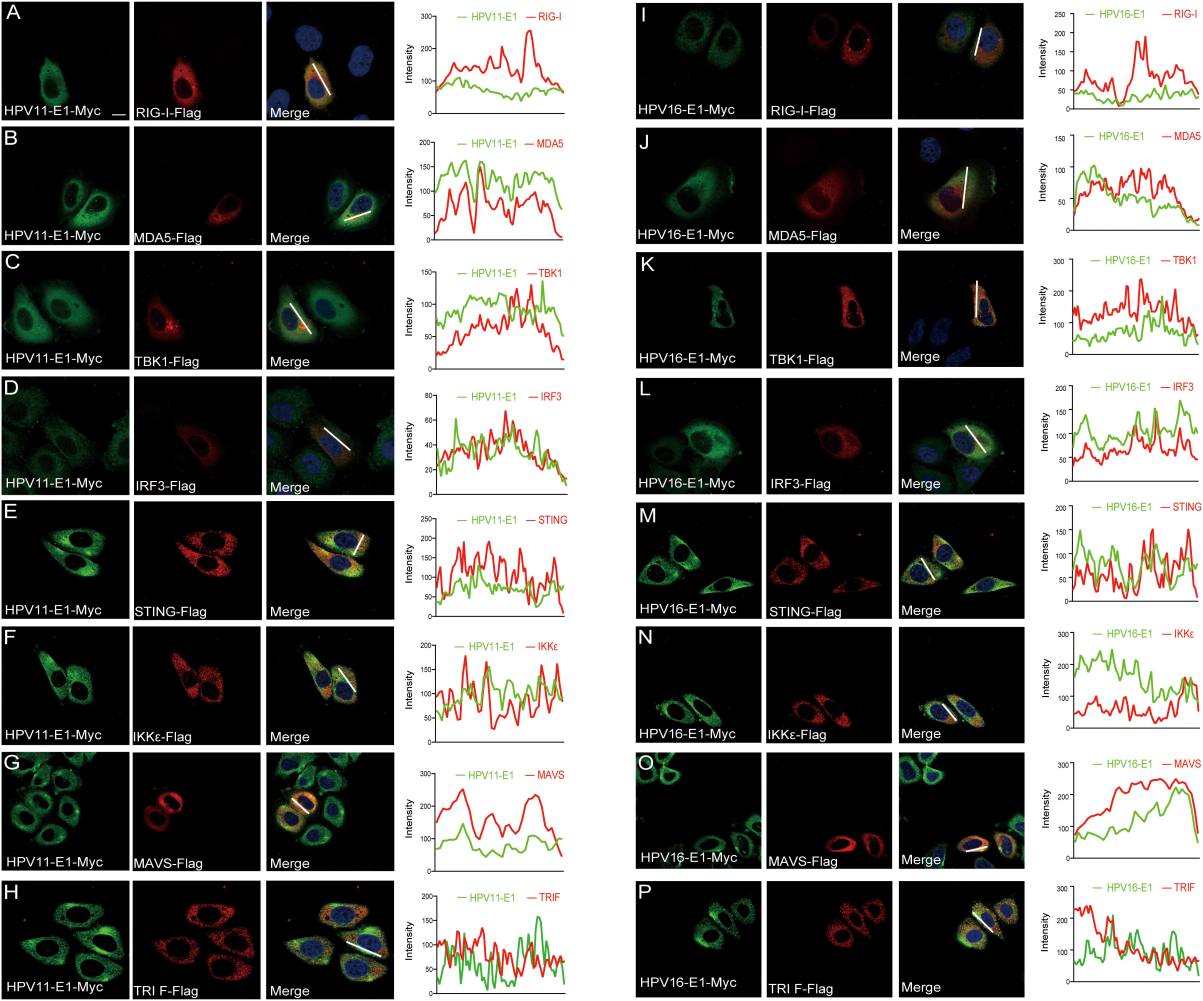
E1 colocalizes with proteins of innate immune signaling pathways. HeLa cells were transfected with plasmids encoding HPV11 E1 **(A-H)** or HPV 16 E1 **(I-P)** and innate immune signaling proteins. After 20 hours, cells were fixed, stained, and imaged using confocal microscopy. Nuclei were stained with DAPI (blue). Scale bar, 10 μm.

To investigate whether HPV E1 interacts with components of these PRR pathways, we performed co-immunoprecipitation and confocal microscopy assays. Co-immunoprecipitation results showed that both HPV11 and HPV16 E1 interacted with RIG-I, MDA5, MAVS, TRIF, STING, IKKϵ, TBK1, and IRF3 (**Figures 5A, B**). Confocal microscopy studies revealed that E1 protein localized in ER (**Figures 6A–F**) and showed co-localization with these signaling proteins (**Figures 7A–P**). These findings suggest that HPV E1 inhibits these pathways by directly binding to key signaling molecules.

### 3.5 HPV E1 represses the phosphorylation and nuclear translocation of IRF3

**Figure 8:**
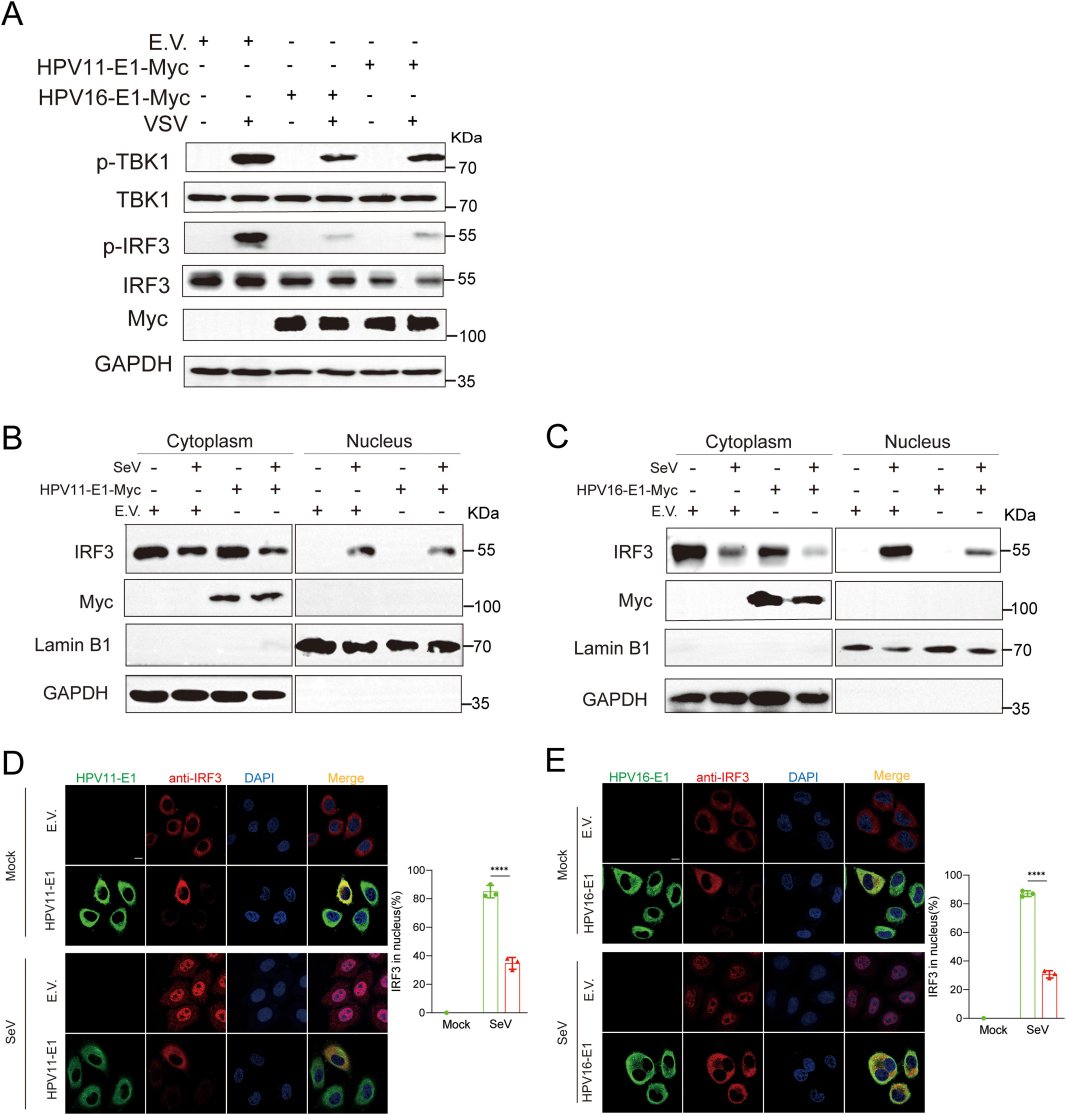
E1 inhibits phosphorylation and nuclear translocation of IRF3. **(A)** HEK293T cells transfected with HPV11 E1, HPV16 E1, or an empty vector were infected with VSV (MOI=0.1) for 6 hours. Western blot analysis was performed to evaluate TBK1 and IRF3 phosphorylation. **(B, C)** Nuclear-cytoplasmic fractionation and Western blotting were conducted on HEK293T cells overexpressing HPV11 E1 or HPV16 E1 following SeV infection (MOI=1) for 6 hours. **(D, E)** Immunofluorescence staining of HeLa cells overexpressing HPV16 E1 or HPV11 E1 reveals IRF3 nuclear translocation following SeV infection. Scale bar, 10 μm. The percentage of IRF3 translocating to the nucleus (right) was calculated based on 100 cells per group in three independent replicates. Statistical significance is shown in the figure. Statistical significance was analyzed using Student’s t-test (**** p < 0.0001).

TBK1 phosphorylation is a critical convergent step in the downstream signaling of the RIG-I/MDA5-MAVS, TLR3-TRIF, and cGAS-STING pathways, although each pathway utilizes distinct upstream adaptor proteins. Upon activation, TBK1 undergoes autophosphorylation and recruits IRF3, resulting in IRF3 phosphorylation and its translocation to the nucleus to initiate IFN transcription (50). Here, we found that HPV E1 overexpression in HeLa cells significantly reduced TBK1 and IRF3 phosphorylation, in contrast to the increased phosphorylation observed in VSV-infected control HeLa cells (**Figure 8A**). To determine whether HPV E1 affects IRF3 nuclear translocation, nuclear-cytoplasmic fractionation and confocal microscopy were performed. The results indicated that, although IRF3 translocates from the cytoplasm to the nucleus in response to SeV infection, HPV E1 expression inhibits this nuclear translocation, retaining IRF3 in the cytoplasm (**Figures 8B–E**). Collectively, these findings demonstrate that HPV E1 inhibits both the phosphorylation and nuclear translocation of IRF3 during viral infection.

### 3.6 HPV E1 suppress the activation of JAK-STAT pathway

**Figure 9:**
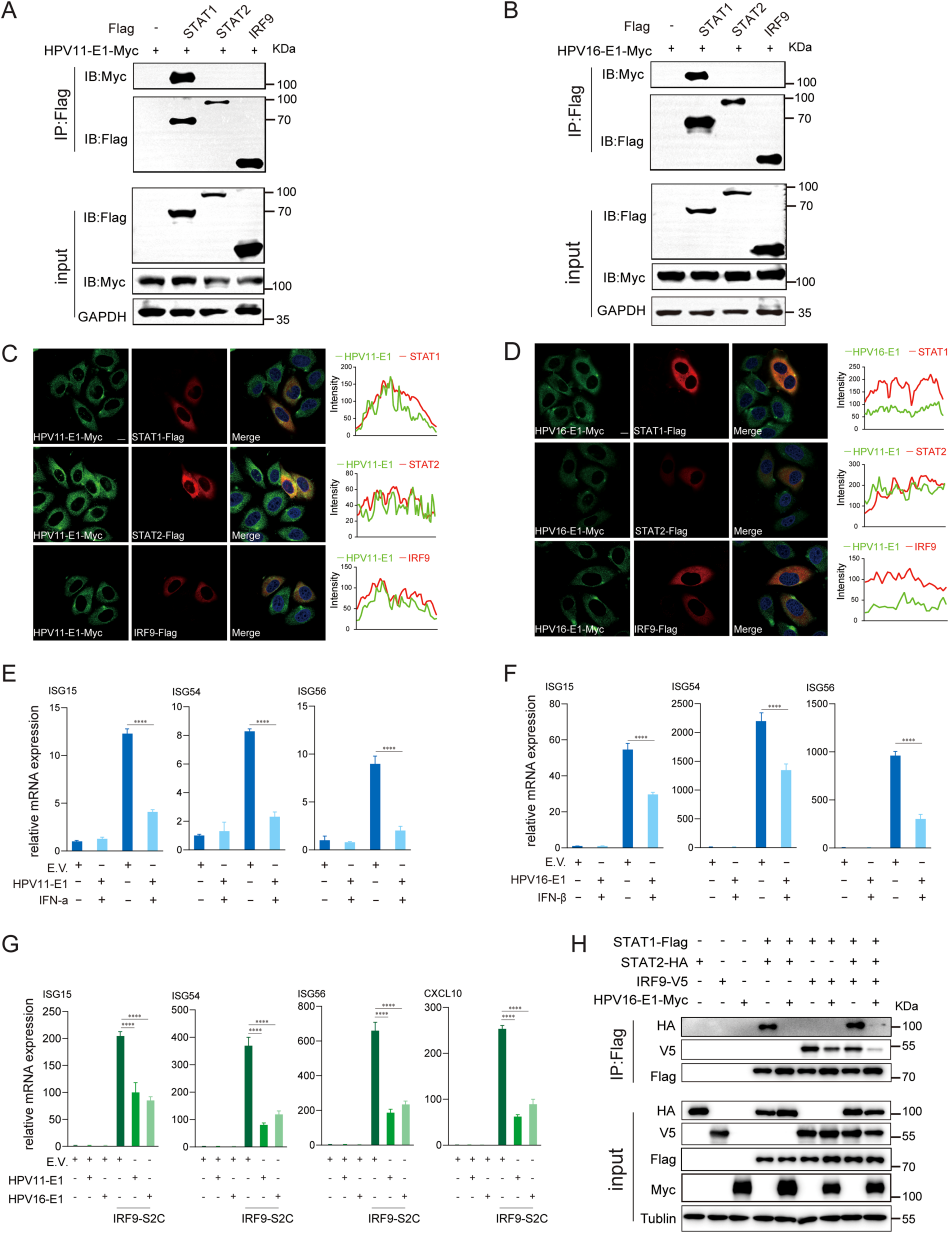
E1 suppresses JAK-STAT pathway and inhibits ISGF3 complex formation. **(A, B)** HEK293T cells co-transfected with HPV11 E1 **(A)** or HPV16 E1 **(B)** and STAT1, STAT2, or IRF9 plasmids were analyzed by co-immunoprecipitation to assess protein interactions 36-48 hours post-transfection. **(C, D)** HeLa cells transfected with HPV11 E1 or HPV16 E1 and STAT1, STAT2, or IRF9 were visualized using confocal microscopy. Nuclei were stained with DAPI (blue). Scale bar, 10 μm. **(E, F)** RT-qPCR analysis of ISG expression in HEK293T cells transfected with E1 or an empty vector and stimulated with IFN-α or IFN-β. **(G)** RT-qPCR analysis of ISG expression in HEK293T cells co-transfected with E1 and IRF9-S2C plasmids. **(H)** Co-immunoprecipitation was performed to evaluate interactions between HPV16 E1 and STAT1, STAT2, and IRF9 in HEK293T cells. Statistical significance was analyzed using Student’s t-test (**** p < 0.0001).

IFNs, generated through the activation of the RIG-I/MDA5-MAVS, TLR3-TRIF, and cGAS-STING pathways, bind to IFNAR to activate the downstream JAK-STAT pathway, thereby amplifying host antiviral response (51). However, whether HPV E1 affects the activation of the JAK-STAT pathway remains unclear. To address this, we analyzed the interaction and colocalization of HPV E1 with STAT1, STAT2, and IRF9 through confocal microscopy and co-immunoprecipitation. Confocal microscopy studies revealed that both HPV11 and HPV16 E1 colocalize with STAT1, STAT2, and IRF9, but exhibited a particular interaction with STAT1 in co-immunoprecipitation assays (**Figures 9A–D**). Upon activation of JAK-STAT pathway, STAT1, STAT2, and IRF9 assemble into the ISGF3 complex, which translocates to the nucleus to drive ISG transcription. To evaluate the effect of HPV E1 on this process, HEK293T cells were treated with IFN-α and IFN-β to stimulate ISG expression. HPV16 E1 expression significantly inhibited the induction of ISG15, ISG54, and ISG56 compared to control cells (**Figures 9E, F**). Furthermore, co-transfection with an IRF9-S2C plasmid, which directly activates ISGF3-mediated transcription, demonstrated that both HPV11 and HPV16 E1 markedly inhibited ISG15, ISG54, ISG56, and CXCL10 expression (**Figure 9G**). Co-immunoprecipitation analysis confirmed that, while STAT1, STAT2, and IRF9 interact with each other in HEK293T cells, HPV16 E1 disrupts these interactions, thereby preventing ISGF3 complex assembly (**Figure 9H**). These observations suggest that HPV E1 suppresses JAK-STAT signaling by disrupting ISGF3 complex formation.

### 3.7 HPV E1 blocks nuclear translocation of ISGF3 complex

**Figure 10:**
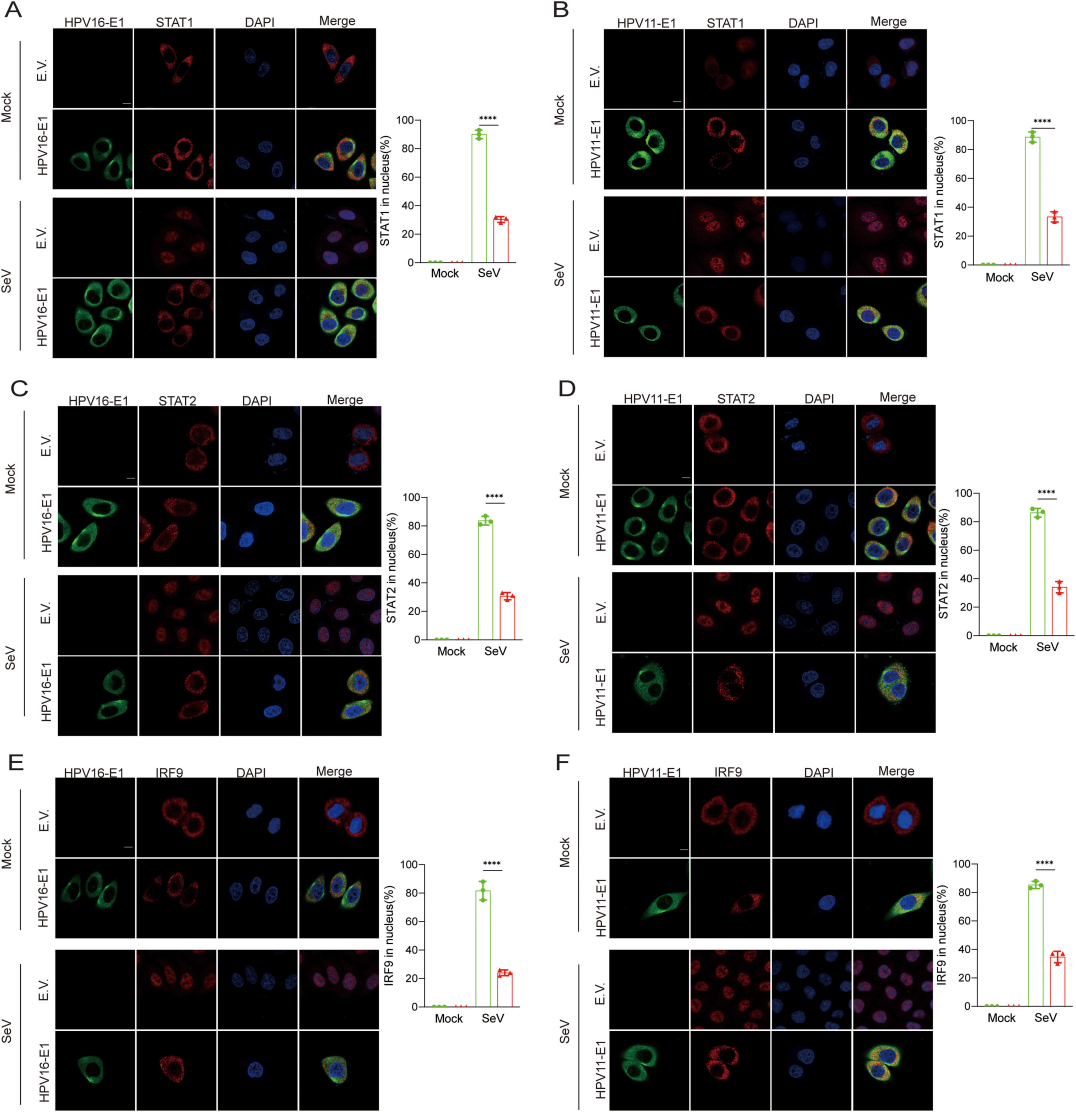
Confocal analysis of E1’s inhibition of nuclear translocation of STAT1, STAT2, and IRF9. Representative images showing the nuclear translocation of STAT1 **(A, B)**, STAT2 **(C, D)**, and IRF9 **(E, F)**. HeLa cells were transfected with plasmids expressing HPV11 E1 or HPV16 E1 along with plasmids expressing STAT1, STAT2, or IRF9, while control cells were transfected with an empty vector. Cells were then infected with SeV (MOI=1) for 6 hours, then fixed, blocked, and incubated with primary antibodies followed by fluorescence-labeled secondary antibodies. Anti-Flag antibodies were used to detect STAT1, STAT2, and IRF9, while anti-Myc antibodies were used to detect HPV11 E1 and HPV16 E1. Nuclear were stained with DAPI (blue). Scale bar, 10 mm. The percentage of STAT1, STAT2, and IRF9 translocating to the nucleus in experimental and control group was calculated based on immunofluorescence results post-SeV infection. This analysis was performed on 40-50 cells per group across 3 independent replicates (right). Statistical significance is indicated in the figure (**** p < 0.0001).

**Figure 11:**
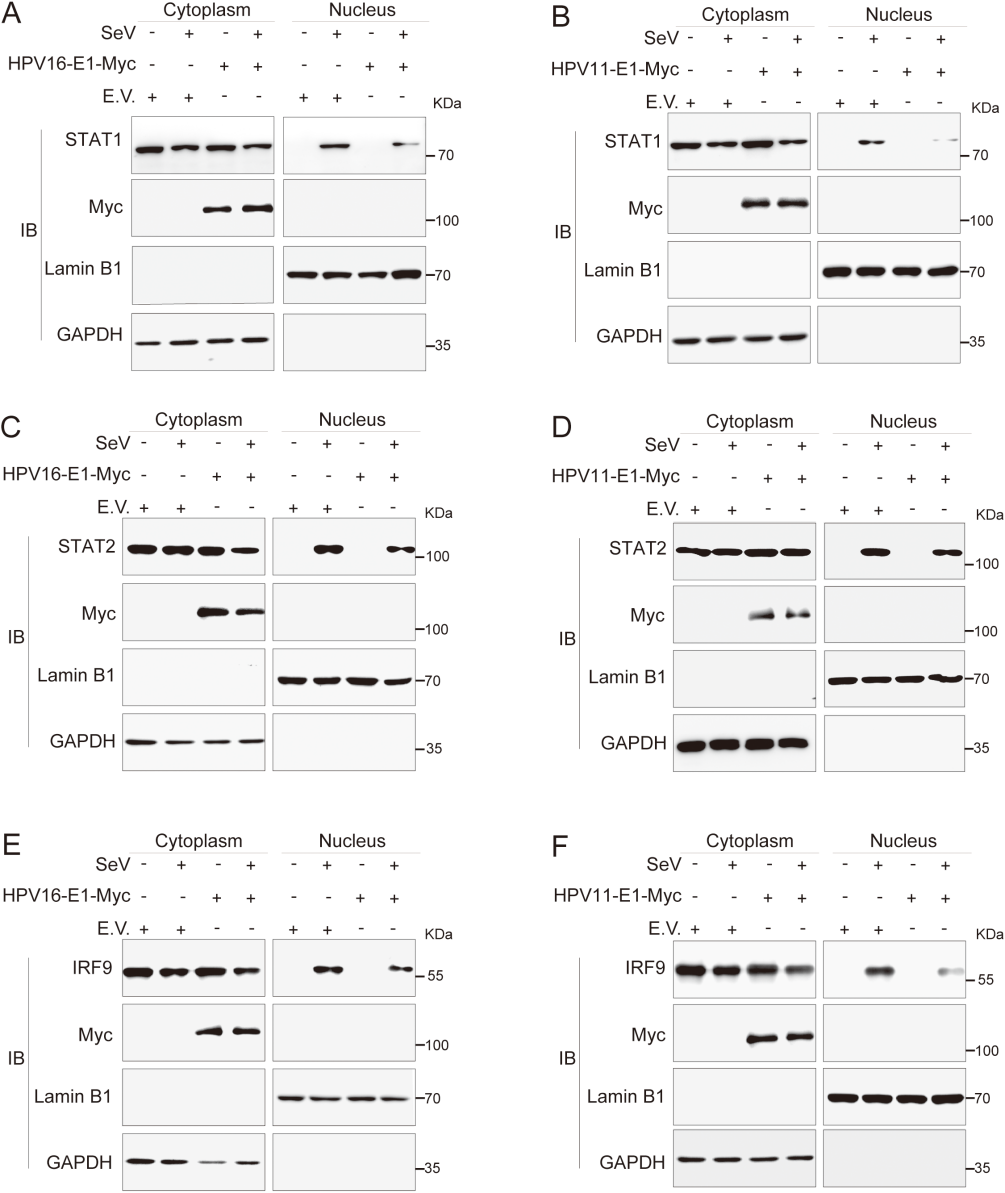
Nuclear-cytoplasmic fractionation analysis of E1-mediated inhibition of nuclear translocation of STAT1, STAT2, and IRF9. HeLa cells were transfected with plasmids expressing HPV11 E1 or HPV16 E1, together with plasmids encoding STAT1 **(A, B)**, STAT2 **(C, D)**, or IRF9 **(E, F)**. Control cells were transfected with an empty vector as indicated. Twenty-four hours after transfection, cells were infected with SeV (MOI = 1) for 6 hours, followed by nuclear-cytoplasmic fractionation and Western blotting as described in **Figures 8B, C**.

Previous findings indicated that the E1 protein suppresses JAK-STAT pathway activation by targeting STAT1 and disrupting interaction within the ISGF3 complex. To further elucidate this mechanism, we examined whether E1 inhibits ISGF3 nuclear translocation. HeLa cells were co-transfected with plasmids encoding HPV E1 and STAT1, STAT2, or IRF9, and nuclear translocation was analyzed using confocal microscopy. Under basal conditions, overexpressed STAT1, STAT2, and IRF9 predominantly localized in the cytoplasm. Following SeV infection, these proteins translocated from the cytoplasm to the nucleus, as anticipated. However, in the presence of E1, the nuclear translocation of STAT1, STAT2, and IRF9 was significantly inhibited (**Figures 10A–F**). Results from nuclear-cytoplasmic fractionation and Western blot analysis further support that E1 blocks SeV-induced ISGF3 nuclear translocation (**Figures 11A–F**). This retention inhibits ISGF3 from initiating ISG transcription, revealing another mechanism through which HPV E1 suppresses the host antiviral response.

## 4 Discussion

**Figure 12:**
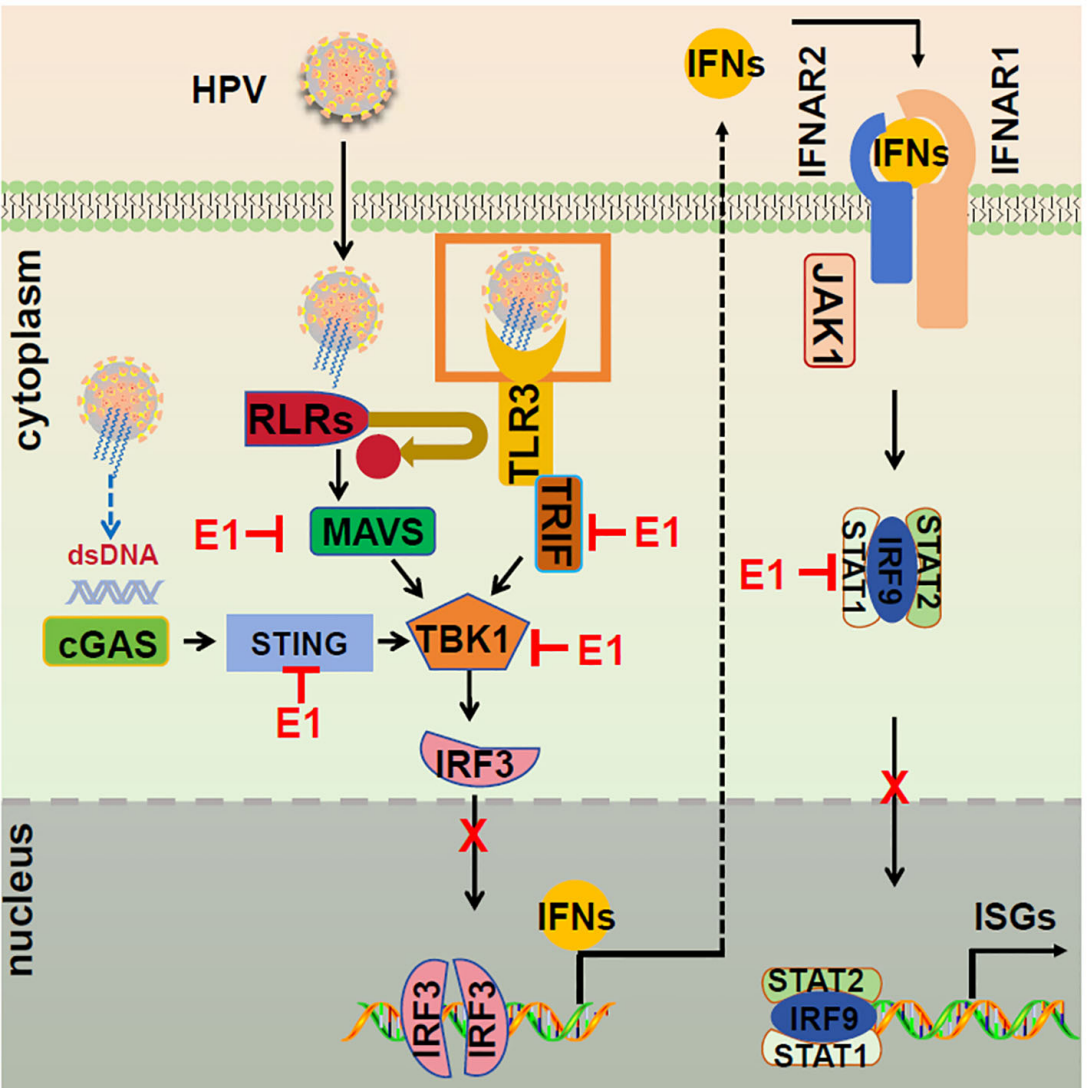
Proposed model of E1 suppressing of IFN responses. The RIG-I/MDA5-MAVS, TLR3-TRIF, cGAS-STING, and JAK-STAT signaling pathways are activated following viral infections. During HPV infection, E1 disrupts these pathways by interacting with RIG-I/MDA5, TRIF, and STING, inhibiting TBK1 and IRF3 phosphorylation, and preventing IRF3 nuclear translocation, resulting in suppressed IFN induction. Additionally, E1 interacts with STAT1, hindering ISGF3 complex formation and nuclear translocation, thereby suppressing ISG transcription and promoting viral replication.

The HPV E1 protein, traditionally recognized for its role in viral DNA replication (34), is shown here to also modulate host immune responses (40, 41). We specifically demonstrate that the HPV11 and HPV16 E1 proteins suppress multiple innate immune signaling pathways, including RIG-I/MDA5-MAVS, TLR3-TRIF, cGAS-STING, and JAK-STAT, thereby inhibiting the production of type I IFNs and ISGs (**Figure 12**). This immune evasion strategy likely facilitates viral persistence and enhances HPV’s capacity to evade host immune surveillance. These findings broaden our understanding of HPV E1, highlighting its role as both a replication factor and an immune suppressor across HR and LR HPV strains.

Our study demonstrates that HPV16 E1 suppresses IFN and ISG responses against diverse stimuli, including poly(I:C), VSV, HSV1, MHV, and HPV16 (**Figures 1A–E**), suggesting broad-spectrum inhibition of immune activation. While prior studies on HPV8 E1 reported diminished poly(I:C)-induced type I IFN and CXCL10 secretion (52), the molecular mechanisms underlying these effects remain poorly understood. Our results provide mechanistic insights, demonstrating that HPV16 and HPV11 E1 proteins interact with signaling molecules such as RIG-I, MDA5, MAVS, TRIF, and STING, thereby inhibiting their pathway-specific functions (**Figures 3A, B**, **4A–C**). Co-immunoprecipitation experiments revealed that both HPV11 and HPV16 E1 proteins interact with RIG-I, MDA5, and MAVS, effectively disrupting the formation of the RIG-I/MDA5-MAVS complex (**Figures 5A, B**). E1 also interacts with TRIF and STING, thereby preventing the formation of the TRIF/STING-TBK1 complex (**Figures 5A, B**). Additionally, E1 interacts with TBK1, IRF3(5D), and IKKϵ, further inhibiting the signaling transduction in these innate immune pathways (**Figures 5A, B**). Notably, HPV16 E1 effectively inhibits the phosphorylation and nuclear translocation of IRF3, a process critical for type I IFN production (**Figures 8A, C, E**). This mirrors immune evasion strategies used by other HPV proteins, such as E6 and E7, which inhibit IRF3 (53) and STING function (49), respectively, highlighting E1’s role as an immune modulator alongside E6 and E7.

Although TLR3-TRIF and RIG-I/MDA5-MAVS are primarily RNA-sensing pathways, emerging evidence also suggests that they also play a role in HPV infection (29). HPV encodes several proteins that interfere with these pathways. For example, E5 impairs TLR3-TRIF signaling, while E6 downregulates TLR3 expression, thereby limiting immune detection (32, 33). Previous studies primarily focused on HPV E6 and E7 proteins in immune evasion, targeting the RLR (33), TLR (54, 55), cGAS-STING (32), and JAK-STAT pathways (56, 57). E6, for example, suppresses RIG-I-mediated signaling by binding to TRIM25, thereby inhibiting K63-linked ubiquitination of RIG-I and reducing its interaction with MAVS (33). In contrast, E7 promotes STING degradation, disrupting cGAS-STING signaling and diminishing type I IFN production (32). Our study expands this understanding by demonstrating that E1 employs unique mechanisms to target multiple upstream components of innate immune pathways, indicating a broader suppression of PRR signaling not previously linked to HPV E1 proteins. A novel discovery in this study is E1’s ability to inhibit TBK1 and IRF3 phosphorylation, effectively suppressing downstream immune activation and type I IFN production. This mechanism underscores E1’s distinctive role in HPV immune evasion, expanding our understanding beyond the traditional roles of E5, E6, and E7. In contrast to E6 and E7, which target specific nodes within immune pathways, E1 acts as a multi-pathway inhibitor, thereby enhancing HPV’s immune evasion capabilities and supporting persistent infection. Additionally, while E1 is primarily known for its role in viral replication, its immunosuppressive functions may indirectly contribute to carcinogenesis. By dampening innate immune activation, E1 could create a cellular environment that supports the oncogenic activities of E5, E6, and E7, all of which drive malignant transformation. This potential interplay suggests that E1 may act as an early facilitator of HPV-induced oncogenesis, warranting further investigation into its contribution to disease progression.

Regarding the JAK-STAT pathway, our data show that both HPV11 and HPV16 E1 suppress ISG expression induced by IFN-α and IFN-β (**Figures 9E, F**). Further analysis reveals that HPV11 and HPV16 E1 attenuate ISGF3-induced ISG expression, as confirmed using an IRF9-S2C plasmid specifically designed to activate ISG transcription (**Figure 9G**). Co-immunoprecipitation and confocal microscopy assays demonstrated an interaction between HPV11 and HPV16 E1 and STAT1 (**Figures 9B, D**), a critical component of the ISGF3 complex. This interaction disrupts ISGF3 complex formation, preventing the nuclear translocation of STAT1, STAT2, and IRF9, and thereby inhibiting ISG transcription (**Figures 9H**, **10**). This suppression mechanism highlights E1’s multifaceted role as a broad immune evasion factor. By inhibiting ISGF3 complex formation, E1 markedly impairs the host’s antiviral response. Previous studies on HPV E7 reported similar inhibition of the JAK-STAT pathway via disruption of ISGF3 complex formation (58, 59); however, our findings identify a distinct mechanism for HPV11 and HPV16 E1 involving direct interaction with STAT1. This adds a new dimension to our understanding of how HPV proteins modulate the host immune system.

Furthermore, our findings reveal that HPV16 E1 exerts dual inhibitory effects by targeting both upstream IFN production pathways (RIG-I/MDA5-MAVS, TLR3-TRIF, and cGAS-STING) and the downstream IFN response pathway (JAK-STAT). This broad-spectrum suppression of immune signaling allows the virus to evade detection at multiple stages, promoting viral persistence. The ability of HPV16 E1 to target critical nodes in immune responses underscores its potential as a therapeutic target. Future studies should investigate strategies to block the interaction between HPV16 E1 and STAT1 or other key components in these pathways, potentially restoring innate immune function and enhancing antiviral defenses against HPV-related diseases.

Despite these significant findings, our study has certain limitations. Primarily, our experiments were conducted *in vitro* using cell lines, which may not fully capture the complexity of HPV-host interactions *in vivo*. The immune microenvironment in tissues, where diverse immune cell interactions take place, likely plays a critical role in shaping viral persistence and immune evasion. To confirm these findings and establish their physiological relevance, future research should utilize animal models and patient-derived tissue samples. Moreover, although investigating HPV E1’s role in immune evasion using E1-deficient viruses might seem like a viable approach, this is technically unfeasible. E1 is essential for the initial amplification of the viral genome, and without it, the virus cannot establish replication, making the generation of viable E1-deficient HPV impossible. Given this constraint, experimental strategies relying on such viruses are not feasible, and no HPV study to date has employed E1-deficient viruses for functional investigations. Additionally, while we identified critical interactions between HPV E1 and multiple immune signaling components, the precise molecular mechanisms underlying these interactions remain unclear. It is uncertain whether E1 directly modulates post-translational modifications of key immune signaling proteins or whether other host factors are involved. Future research should aim to characterize these specific molecular interactions and assess whether E1-mediated immune suppression is conserved across different HPV types. Comparative studies across HPV genotypes will be critical to determine whether E1 serves as a universal suppressor of immune responses, with profound implications for developing therapeutic strategies targeting E1 in HPV-related diseases.

## 5 Conclusion

In summary, this study identifies a previously uncharacterized role for HPV E1 as broad-spectrum suppressors of the host innate immune response. By targeting multiple critical signaling pathways, including RIG-I/MDA5-MAVS, TLR3-TRIF, cGAS-STING, and JAK-STAT, E1 promotes viral persistence and facilitates immune evasion (**Figure 12**). These findings enhance our understanding of HPV’s immune evasion strategies and suggest novel therapeutic approaches to restore innate immune signaling for combating HPV infections.

## Data Availability

The raw data supporting the conclusions of this article will be made available by the authors, without undue reservation.
