# Supplementary material for: Human papillomavirus E1 proteins inhibit RIG-I/MDA5-MAVS, TLR3-TRIF, cGAS-STING, and JAK-STAT signaling pathways to evade innate antiviral immunity
Source: Front Immunol. 2025 Apr 22;16:1549766. doi: 10.3389/fimmu.2025.1549766 (PMC12052760; doi:10.3389/fimmu.2025.1549766)
Supplement: Supplementary file 1 [file DataSheet1.docx]

**Supplementary materials**

**Human Papillomavirus E1 Proteins Inhibit RIG-I/MDA5-MAVS, TLR3-TRIF, cGAS-STING, and JAK-STAT Signaling Pathways to Evade Innate Antiviral Immunity**

Jin-Xin Li ^1^, Jing Zhang ^1^, Cheng-Hao Li ^2^, Yun-Fang Li ^2^, Hui-Min Chen ^2^, Tao Li ^1^, Qing Zhang ^3, 4^, Bei-Hua Kong ^3, 4^, Pei-Hui Wang ^1, 2, *^

^1^ Department of Infectious Disease and Hepatology, The Second Hospital of Shandong University, Cheeloo College of Medicine, Shandong University, Jinan, 250033, Shandong, China;

^2^ Key Laboratory for Experimental Teratology of Ministry of Education and Advanced Medical Research Institute, Cheeloo College of Medicine, Shandong University, Jinan, Shandong 250012, China.

^3^ Department of Obstetrics and Gynecology, Qilu Hospital, Shandong University, 107 Wenhua Xi Road, Jinan, 250012, China.

^4^ Gynecologic Oncology Key Laboratory of Shandong Province, Qilu Hospital, Shandong University, Jinan, 250012, China.

*Correspondence: [pei-hui.wang@connect.hku.hk](mailto:pei-hui.wang@connect.hku.hk)

**Supplementary Table S1. Primers used in this study**

| Primer name | Sequence (5′-3′) | Usage |
| --- | --- | --- |
| GAPDH-F  GAPDH-R | GGAGCGAGATCCCTCCAAAAT  GGCTGTTGTCATACTTCTCATGG | RT-qPCR |
| IFN-β-F  IFN-β-R | TTGCTCTCCTGTTGTGCTTC  AAGCCTCCCATTCAATTGCC | RT-qPCR |
| ISG56-F  ISG56-R | CTAAGCAAAACCCTGCAGAAC  TCAGGCATTTCATCGTCATC | RT-qPCR |
| ISG54-F  ISG54-R | CGAACAGCTGAGAATTGCAC  TTCTCCCTCCATCAAGTTCC | RT-qPCR |
| CXCL10-F  CXCL10-R | GTGGCATTCAAGGAGTACCTC  GACCTTTCCTTGCTAACTGCT | RT-qPCR |
| ISG15-F | AGGCAGCGAACTCATCTTTG | RT-qPCR |
| ISG15-R | GGACACCTGGAATTCGTTG |  |
| HPV16 E1-F | CTTGGTACCGAGCTCGCCACCATGGCCGACCCTGCCGGAA | ORF cloning |
| HPV16 E1-R | CACGGTGTTGTCCTTTCTAGACAGGGTGTTGGTGTTCTGGCCG |  |
| HPV11 E1-F | CTTGGTACCGAGCTCGCCACCATGGCCGACGACAGCGGCA | ORF cloning |
| HPV11 E1-R | CACGGTGTTGTCCTTTCTAGACAGGGTTCTCACCACGCTGCCA |  |

F: forward primer. R: reverse primer.


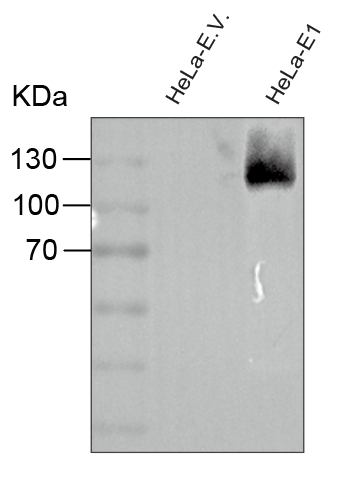


**Supplementary Figure S1. HeLa cells stably expressing HPV16 E1 or an empty vector were lysed, and Western blot analysis was performed to assess protein expression.**

> **The IFNβ promoter regions cloned into the pGL3 vector**

CTAACTTCACAGAGCTCCCTCTTCTGTTAGCTTTTGTGAAATGGTCAAAAACATAGCAGCCTGCCTTCTGAGTTCTCCATCCCACCCTGGTTGGGCCTTCTCTATCCTTGTCTCTGTTGTTTATATCCTGCTGAAGTGTGATTCCACTTGTGGCAGTTTCTCCTCTGTGTAGGATCAAAAGGGCTGTGGCTGGTTGGTTTGAAAATTTCTTATACCCTAGACTATTCCAGTGCCTTTCAGAAGTTTCCAAGGCCCTCTCACACTAATCTATTATCATATTGGGCAAAACTCCTTGCAGTTTCAGCTACTATTCCCTGATTGACTTTTCAGTAAATCTATCTCTCAGTCTTTCAGTATCCAAAGAAGATTGGTTCTAGGACCACCATCCCGCTGCCTCCACAGATACCAAAATCAGAGGATGCTCAATTCCCTCTTATAAAACGTTGCAGTATTTGCATATAATCTGCACATGTATTTCTGTATATTTTAAATCATCCCTAGATTACTTATAATACCTGATACAATATAAATGCTAAATAGCTGTAACACTGTATCTTTAAAATTTACATTATTTTTTGTTGTTGTATTATTATTTTTATTGTATTTTTAAAAAATATTTTCCATCTACAGTCAGTAGAATCCACGGATACAGAACCTATGGATAGGAAGGACCAACTGTATCTTTTAGTGTTTTGAGGTTCTTGAATTCTCAGGTCGTTTGCTTTCCTTTGCTTTCTCCCAAGTCTTGTTTTACAATTTGCTTTAGTCATTCACTGAAACTTTAAAAAACATTAGAAAACCTCACAGTTTGTAAATCTTTTTCCCTATTATATATATCATAAGATAGGAGCTTAAATAAAGAGTTTTAGAAACTACTAAAATGTAAATGACATAGGAAAACTGAAAGGGAGAAGTGAAAGTGGGAAATTCCTCTGAATAGAGAGAGGACCATCTCATATAAATAGGCCATACCCATGGAGAAAGGACATTCTAACTGCAACCTTTCGAAGCCTTTGCTCTGGCACAACAGGTAGTAGGCGACACTGTTCGTGTTGCAAC

> **The sequences for the codon-optimized HPV16 E1**

ATGGCCGACCCTGCCGGAACAAACGGCGAGGAGGGCACAGGCTGCAACGGCTGGTTTTACGTGGAGGCCGTGGTGGAGAAGAAGACAGGCGATGCCATCAGCGACGATGAGAATGAGAACGATAGCGATACCGGCGAGGATCTGGTGGATTTTATCGTGAACGACAATGACTACCTGACCCAGGCCGAGACCGAGACCGCCCACGCTCTGTTTACCGCCCAGGAGGCCAAGCAGCACAGGGACGCCGTGCAGGTGCTGAAGAGAAAGTACCTGGGCAGCCCACTGTCCGACATCAGCGGCTGTGTGGACAACAACATCAGCCCTAGACTGAAGGCCATCTGTATCGAGAAGCAGAGCAGAGCCGCCAAGAGGAGGCTGTTCGAGTCCGAGGACTCCGGCTACGGCAACACAGAGGTGGAGACACAGCAGATGCTGCAGGTGGAGGGCAGACACGAGACAGAGACACCCTGTAGCCAGTACTCCGGCGGCAGCGGCGGCGGATGTTCCCAATACAGCTCCGGCTCCGGCGGCGAGGGCGTTTCTGAGAGGCACACCATCTGCCAGACACCCTTGACCAACATCCTGAATGTGCTGAAGACATCCAACGCCAAGGCCGCCATGCTGGCCAAGTTCAAGGAGCTGTACGGCGTGAGCTTTAGCGAGCTGGTGAGACCCTTCAAGAGCAATAAGTCCACATGTTGTGATTGGTGCATCGCCGCCTTTGGCCTGACACCCAGCATCGCCGATTCCATCAAGACACTGCTGCAGCAGTACTGTCTGTACCTGCACATCCAGTCCCTGGCCTGCTCCTGGGGCATGGTGGTGCTGCTGCTGGTGAGGTACAAGTGTGGCAAGAATAGGGAGACCATCGAGAAGCTGCTGAGCAAGCTGCTGTGCGTGAGCCCTATGTGCATGATGATCGAGCCTCCCAAGCTGAGAAGCACCGCCGCCGCCCTGTACTGGTACAAGACAGGCATCTCCAATATCAGCGAGGTGTACGGCGACACACCTGAGTGGATTCAAAGACAGACCGTGCTGCAGCACAGCTTCAACGACTGCACATTCGAGCTGAGCCAGATGGTGCAGTGGGCCTACGACAATGATATTGTGGACGACAGCGAGATCGCCTACAAGTACGCCCAGCTGGCCGATACAAATAGCAATGCCAGCGCCTTTCTGAAGAGCAATTCCCAGGCCAAGATCGTGAAGGATTGTGCCACAATGTGTAGACACTACAAGAGAGCCGAGAAGAAGCAGATGTCCATGAGCCAGTGGATCAAGTACAGATGCGATAGGGTGGACGACGGCGGCGATTGGAAGCAGATCGTGATGTTTCTGAGATACCAGGGCGTGGAGTTCATGAGCTTTCTGACAGCCCTGAAGAGGTTCCTGCAGGGCATCCCAAAGAAGAACTGTATCCTGCTGTACGGCGCCGCCAACACAGGCAAGTCCCTGTTCGGCATGTCCCTGATGAAGTTTCTGCAGGGCTCCGTGATCTGCTTCGTGAATAGCAAGTCCCACTTTTGGCTGCAGCCTCTGGCCGATGCCAAGATCGGCATGCTGGATGATGCCACCGTGCCTTGCTGGAATTACATCGACGACAACCTGAGGAATGCCCTGGATGGCAATCTGGTGTCCATGGATGTGAAGCACAGGCCCTTGGTGCAGCTGAAGTGTCCTCCCTTGCTGATCACCAGCAACATCAATGCCGGCACAGACTCCAGATGGCCCTACCTGCACAATAGGCTGGTGGTGTTCACATTCCCTAACGAGTTCCCTTTTGATGAGAATGGCAACCCAGTGTACGAGCTGAATGATAAGAACTGGAAGTCCTTTTTTTCCAGGACATGGTCCAGACTGAGCCTGCACGAGGATGAGGACAAGGAGAACGACGGCGACAGCCTGCCTACATTCAAGTGCGTGTCCGGCCAGAACACCAACACCCTG

> **The sequences for the codon-optimized HPV11 E1**

ATGGCCGACGACAGCGGCACCGAGAATGAGGGCAGCGGCTGTACAGGCTGGTTCATGGTGGAGGCCATCGTGGAGCACACCACCGGCACACAGATCTCCGAGGATGAGGAGGAGGAGGTGGAGGACAGCGGCTACGATATGGTGGATTTCATCGACGATAGACACATCACCCAGAACAGCGTGGAGGCCCAGGCCCTGTTCAATAGGCAGGAGGCCGACGCCCACTACGCCACAGTGCAGGATCTGAAGAGAAAGTACCTGGGCAGCCCATACGTGAGCCCTATCTCCAACGTGGCCAACGCCGTGGAGAGCGAGATCAGCCCTAGACTGGACGCCATCAAGCTGACAACACAGCCCAAGAAGGTGAAGAGAAGACTGTTCGAGACAAGGGAGCTGACCGACTCCGGCTACGGCTACTCCGAGGTGGAGGCCGCCACACAGGTGGAGAAGCACGGCGATCCCGAGAATGGCGGCGATGGCCAGGAGAGAGATACAGGCAGGGACATCGAGGGCGAGGGCGTGGAGCACAGGGAGGCTGAGGCCGTGGATGACTCCACCAGGGAGCACGCCGACACCTCCGGCATCCTGGAGCTGCTGAAGTGCAAGGACATCAGATCCACCCTGCACGGCAAGTTCAAGGATTGTTTTGGCCTGTCCTTCGTGGATCTGATCAGGCCTTTCAAGTCCGATAGGACCACCTGTGCCGATTGGGTGGTGGCCGGCTTCGGCATCCACCACAGCATCGCCGACGCCTTTCAGAAGCTGATCGAGCCCTTGAGCCTGTACGCCCACATCCAGTGGCTGACCAATGCCTGGGGCATGGTGCTGCTGGTGCTGATCAGGTTCAAGGTGAATAAGAGCAGATGCACCGTGGCCAGAACACTGGGCACCCTGCTGAACATCCCAGAGAACCACATGCTGATCGAGCCTCCTAAGATCCAGTCCGGCGTGGCCGCCCTGTACTGGTTTAGAACCGGCATCAGCAACGCCTCCACCGTGATCGGCGAGGCTCCTGAGTGGATCACAAGACAGACAGTGATCGAGCACTCCCTGGCCGATTCCCAGTTTAAGCTGACCGAGATGGTGCAGTGGGCCTACGACAATGATATTTGTGAGGAGTCCGAGATCGCCTTCGAGTACGCCCAGAGGGGCGATTTCGACTCCAACGCCAGGGCCTTTCTGAACAGCAACATGCAGGCCAAGTACGTGAAGGATTGCGCCATCATGTGTAGACACTACAAGCACGCCGAGATGAAGAAGATGTCCATCAAGCAGTGGATCAAGTACAGAGGCACCAAGGTGGACTCCGTGGGCAACTGGAAGCCTATCGTGCAGTTTCTGAGGCACCAGAATATCGAGTTCATCCCTTTTCTGTCCAAGCTGAAGCTGTGGCTGCACGGCACACCTAAGAAGAACTGTATCGCCATCGTGGGCCCACCTGACACAGGCAAGAGCTGCTTTTGTATGTCCCTGATCAAGTTTCTGGGCGGCACAGTGATCTCCTACGTGAATAGCTGTTCCCACTTCTGGCTGCAGCCCTTGACAGATGCCAAGGTGGCCCTGCTGGACGACGCCACCCAGCCATGCTGGACATACATGGATACCTACATGAGGAATCTGCTGGACGGCAACCCAATGAGCATCGACAGAAAGCACAGGGCCCTGACACTGATCAAGTGCCCACCTCTGCTGGTGACATCCAATATCGACATCTCCAAGGAGGAGAAGTACAAGTACCTGCACTCCAGGGTGACCACCTTCACATTCCCTAACCCATTCCCATTCGACAGGAACGGCAACGCCGTGTACGAGCTGTCCGACGCCAACTGGAAGTGCTTCTTTGAGAGACTGAGCAGCAGCCTGGATATTGAGGACTCCGAGGATGAAGAGGACGGCAGCAATTCCCAGGCCTTCAGATGTGTGCCTGGCAGCGTGGTGAGAACCCTG
